# Ozone treatment regulated the anti-exercise fatigue effect of fresh-cut pitaya polyphenol extracts

**DOI:** 10.3389/fnut.2024.1500681

**Published:** 2024-11-25

**Authors:** Chen Li, Zhaoxia Wu, Xiaofei Yang

**Affiliations:** ^1^College of Food and Health, Jinzhou Medical University, Jinzhou, China; ^2^College of Food Science, Shenyang Agricultural University, Shenyang, China; ^3^College of Grain Science and Technology, Shenyang Normal University, Shenyang, China

**Keywords:** pitaya polyphenol, ozone, exercise-induced fatigue, gut microbiota, oxidative stress

## Abstract

The anti-exercise fatigue effect of plant polyphenols is closely related to the content, composition, and extractability of phenolic substances. According to our previous findings, ozone treatment significantly affected the biological effects of fresh-cut pitaya polyphenols. Therefore, this study used C57BL/6 J mice to explore the regulatory mechanism of ozone treatment on the anti-exercise fatigue effect of fresh-cut pitaya polyphenol extract. The result showed that fresh-cut pitaya polyphenols treated with ozone (OP) have a better effect. Compared with the untreated group, the exhaustion time of the OP group was 11.86% longer, the total antioxidant capacity was 54.17% higher, the MDA content was 32.8% lower, and the liver glycogen content was 85.71% higher. The OP group also better regulated substrate metabolism, protecting muscle and visceral weight. The results of RT-PCR were consistent with the results of network pharmacology, and the expression of PI3K and AKT was activated, while SRC and STAT3 were down-regulated. Furthermore, pitaya polyphenols have been demonstrated to exert anti-fatigue effects by increasing the diversity of gut microbiota species. In summary, ozone treatment is a feasible way to improve the nutrition of polyphenols.

## 1 Introduction

Exercise-induced fatigue is a physiological phenomenon that occurs when the human body continues to be active mentally or physically to a certain stage (1, 2). Persistent sports fatigue can easily induce sports injuries, which can bring hidden dangers to the body and athletic performance ability of athletes or sports enthusiasts (3–5). Therefore, how to effectively prevent and recover from exercise-induced fatigue currently needs to be considered.

In recent years, research has focused on the development of plant extracts with low toxicity and few side effects as a means of providing natural nutritional supplements for the treatment of human fatigue. The majority of current studies indicate that the source and mechanism of fatigue are related to the exacerbation of oxidative stress, energy levels, substrate depletion, or accumulation of metabolites (6, 7). Excessive fatigue can also lead to gut microbiome disorders, exacerbating the physical damage caused by fatigue. Plant polyphenols have received much attention due to their health-promoting effects. Plant polyphenols are a class of compounds with complex structures, which are highly abundant in fruits and vegetables (8). The ortho phenol hydroxyl group of plant polyphenols is highly susceptible to oxidation, exhibiting a strong affinity for free radicals, including reactive oxygen species. This property has led to the hypothesis that it may possess anti-exercise fatigue properties. At present, the polyphenols and flavonoids of tea (8), hawthorn (9), the seed coat of *Euryale ferox* Salisb (10), and grape seeds (11) have been proven to play an antioxidant and anti-fatigue role by regulating glycogen reserves and aerobic metabolism of energy substances. However, the anti-fatigue effects of most plant polyphenols are still being explored.

Fresh-cut pitaya is rich in polyphenols, which have potential health-promoting effects, and there is a lot of room for research and development in exerting anti-exercise fatigue effects. Nevertheless, the pharmacological and biological effects of plant polyphenols are contingent upon a multitude of variables, which are intricately intertwined with the content, composition, and extractability of phenolic substances (12). The polyphenol content in most fruits and vegetables is not sufficient to exert the expected pharmacological effects of nutritional foods and functional foods. In our previous study, ozone treatment could induce phenolic accumulation and increase phenolic content in fresh-cut pitaya fruit (13, 14). On the other hand, ozone treatment also had a beneficial effect on the bioavailability of polyphenols in fresh-cut pitaya fruit. It is concluded that ozone treatment is closely related to the biological effects of fresh-cut pitaya polyphenols.

In this study, the regulatory mechanism of ozone treatment on the anti-exercise fatigue effect of fresh-cut pitaya polyphenols was investigated from the perspectives of oxidative stress, substrate metabolism, energy metabolism and intestinal flora changes. This study attempted to explore the mechanism of fresh-cut pitaya polyphenols in relieving exercise fatigue through *in vivo* experiments and provided theoretical basis for developing functional foods to improve endurance and relieve fatigue.

## 2 Materials and methods

### 2.1 Plant materials

The red pitaya was procured from its native region (Hainan, China) and harvested at 90% ripeness for use as experimental material. At this stage, the fruit had reached an optimal edible state with a total soluble solid content of approximately 11 to 14%. Following harvest, the fruit was transported via air within 24 h through a cold chain (6°C, 85% relative humidity) to the laboratory. The pitaya was stored in a refrigerator at a temperature of 8 ± 2°C until processing. The fruits were manually peeled and cut into quarter slices (1 cm in thickness), and subsequently randomly packed into polypropylene containers with dimensions of 15 cm × 10 cm × 4 cm.

### 2.2 Ozone treatment

Ozone is produced by corona discharge method (15). After cutting the pitaya fruit, it was immediately exposed to ozone. The sample was placed in ozone gas for ozone exposure (10 μLL^−1^ for 60 min). The control group consisted of untreated fresh-cut pitaya fruit.

### 2.3 Preparation of fresh-cut pitaya polyphenol extracts

The extraction of polyphenols has been adjusted based on pre-experimental results. A total of 100 g pitaya fruit was extracted with 100 mL 80% acetone for 10 min. Mash the mixture and strain with filter paper. Centrifuge 2,500 × g for 10 min. The supernatant evaporates at 45°C after centrifugation. Finally, the volume of extract was fixed to 20 mL with ultrapure water. The polyphenol extract of pitaya fruit was freeze-dried by vacuum and pulverized by ultrafine method for further animal experiments.

### 2.4 Network pharmacology to evaluate the anti-exercise fatigue effect of fresh-cut pitaya polyphenols

#### 2.4.1 Selection of active ingredients and pharmacokinetic evaluation

Based on the results of our previous research, 26 phenolic substances were detected in red pitaya fruit (14). The 26 compounds were subsequently entered into the SWISSADME database^1^ in order to obtain pharmacokinetic data. In this study, a bioavailability score of >0.55 was considered the primary criterion for selecting compounds that play a key role in biological activity. Seventeen compounds met the selection criteria and were used in the subsequent steps (Supplementary Table S1).

#### 2.4.2 Target prediction of phenolic compounds

The PubChem database^2^ can be used to search for and collect information on 17 different functional component-related targets. The active ingredient SMILES can then be input into Swiss Target Prediction^3^ and Similarity of the ensemble approach^4^ to predict drug targets. A script written in the Perl computer programming language is employed for the screening and deduplication of active ingredients and targets, with the name and serial number of the drug targets subsequently displayed. Consequently, the drug ingredient names are converted into gene tags via the Uniprot database.

#### 2.4.3 Construction of exercise fatigue related gene set

Using Gene Card database^5^ and OMIM database^6^ to search and download data of disease targets, to organize and summary.

#### 2.4.4 Screening of functional components and disease targets

Separate the label information from the functional component target file for use and separate the label information corresponding to diseases from Gene Card and other data for use; The R language Venn Diagram package was used to process the da-ta and make the Venn diagram to get the drug-disease corresponding data.

#### 2.4.5 Data visualization and protein–protein interaction (PPI) network construction

The common gene targets of fresh-cut pitaya polyphenols and exercise fatigue were imported into STRING database.^7^ The analysis species was set to “*Homo sapiens*,” the confidence level was set to 0.9, the protein interaction network analysis was performed, and the updated results were downloaded in PNG and TSV format. TSV format files have been imported into Cytoscape software for topology property analysis. According to the topological property analysis results of the above PPI, targets above the median were selected as core targets and screened twice. Based on the above findings, we obtained the core targets in the network.

#### 2.4.6 Enrichment of gene ontology (GO) function and Kyoto encyclopedia of genes and genomes (KEGG) pathway

The requisite data package was installed in R language, the requisite parameters were set, the GO gene function and KEGG pathway were enriched, and the histogram and scatter diagram were generated.

### 2.5 Molecular docking

The analysis was conducted using Swiss Dock. The Pymol software can be employed to remove ligands (if any) and non-protein molecules and save them as PDB suffix files. The small molecules (naringenin, sinapic acid and ferulic acid) of the structure were downloaded from PubChem^8^ in SDF format and then converted to mol2 format using OpenBabel software. The structure files of the target proteins and small molecules can be uploaded to the Swiss Dock platform.^9^

### 2.6 Experimental animals

The seven-week-old male C57BL/6 J mice (21 ± 1 g) were procured from Beijing Huafukang Biotechnology Co., Ltd. The health status of the mice was tested by the Institute of Medical Laboratory Animals, Chinese Academy of Medical Sciences. Animals are transported at room temperature using special transport cartons with breathable grates during which mice are fasted. Upon arrival, mice were subjected to a controlled environment, with a temperature of 24 ± 2°C, humidity of 60 ± 5%, noise level of <50 dB, and a 12/12 h dark/light cycle. It is imperative that a laboratory diet and sterile water be provided throughout the experiment. This study adheres to the ethical standards governing animal experimentation.

### 2.7 Experimental procedures for exercise fatigue mice model

#### 2.7.1 Animal grouping

The mice are allowed to become accustomed to the experimental environment for a period of 1 week. During this period, the mice are trained to swim. Any mice that do not demonstrate the ability to swim are excluded from further participation. The mice that demonstrated the greatest performance were randomly divided into four groups (*n* = 8). The first group was designated the “normal” group (N) and received 200 mg/kg/day of distilled water and no exercise. The second group was designated the “model” group (M) and received 200 mg/kg/day of distilled water and forced exercise. The third group was designated the “polyphenol” group (P), and the fourth group was designated the “ozone-treated polyphenol” group (OP). Both of these groups received 200 mg/kg/day of polyphenol extract and forced exercise. The body weight and food intake of the mice are recorded on a weekly basis. The pitaya polyphenol gavage liquid was prepared by adding 100 mg of polyphenol lyophilized powder to 10 mL of distilled water, which was then shaken at 2000 rpm with a vortex mixer for 5 min.

#### 2.7.2 Forced swim test

The dose of experimental material administered to mice was 200 mg/kg, with the distribution as follows: the control group; the model group; the Group P and the OP group, with all other conditions being identical. The swimming period lasted for 21 days. The body weight and food intake of the mice were recorded at two-day intervals. Adaptive swimming was conducted in a tank measuring 74 cm × 53.5 cm × 41.5 cm at a water temperature of 25.0 ± 1.0°C. This was performed every 3 days, with the first swim lasting 5 min and subsequent swims increasing in duration by 3 min. The final swim was a weight-bearing exercise, with a weight-bearing lead weight of 10% of the mouse’s body weight. At the conclusion of the experiment, the mice were weighed, and their eyeballs were excised. Following cervical dislocation, the organs are separated and weighed. The contents of the mouse colon should be sterilized and stored at −80°C, along with the blood, viscera, mid-thigh skeletal muscle (quadriceps) and intestinal contents.

#### 2.7.3 Biochemical analysis

The mice were euthanized and their eyeballs were removed for the purpose of blood collection. Blood samples were centrifuged at 4000 rpm for 10 min to collect serum samples and stored at −80°C for further experiments. The contents of malondialdehyde (MDA), superoxide dis-mutase (SOD), total antioxidant capacity (T-AOC), glutathione (GSH), lactate dehydrogenase (LDH), creatine kinase (CK), lactate (LD), serum urea nitrogen (BUN) and liver glycogen were measured according to the instructions of the kit.

#### 2.7.4 Hematoxylin and eosin (H&E) staining

For details, refer to the method of Yu et al. (9).

#### 2.7.5 Real-time quantitative PCR analysis

Total RNA was extracted according to the Trizol method, and the concentration and purity of the RNA were determined using a micro-UV spectrophotometer (Boyde Micro Drop). RNA is reverse transcribed to cDNA with a reverse transcription kit (Thermo Scientific Fermentas, K1622) and total RNA is stored in a freezer at −80°C for use, for details, refer to the method of Yu et al. (9).

#### 2.7.6 Detection of microorganisms in intestinal contents

On the 21st day, the cecal contents were collected from the sacrificed mice and stored at a temperature of −80°C for subsequent DNA extraction. The total bacterial genomic DNA was extracted using the Fecal Rapid DNA Spin Kit. The extracted DNA was stored at −80°C and subsequently subjected to 16S rRNA sequencing. Universal primers 341F, 806R, PCR were used to amplify the V3-V4 region of bacterial 16S rRNA gene with high changes. Gel the DNA using a gel/PCR extraction kit, load it, and sequence it. Sequencing results were analyzed using the QIME2 analysis platform and the Silva database was used for species annotation. The diversity of gut microbiota was analyzed using this website^10^ and the effect size was analyzed using the website for linear discriminant analysis (LefSe).^11^

### 2.8 Statistical analysis

Each set of experiments was conducted at least three times in parallel, and the mean was calculated. The final result is expressed as “mean ± standard deviation.” The significance of the Duncan multivariate difference test data was analyzed using SPSS 22.0. *p* < 0.05 was considered statistically significant.

## 3 Results

### 3.1 Results of network pharmacology analysis

#### 3.1.1 Screening of active ingredients of fresh-cut pitaya polyphenols

A total of 17 fresh-cut pitaya polyphenol active ingredients were obtained, including four catechin derivatives, six benzoic acid derivatives, four phenylpropanoids, one flavanone, and two coumarins (Supplementary Table S1).

#### 3.1.2 Identification of potential targets for anti-fatigue effects of pitaya polyphenols

**Figure 1:**
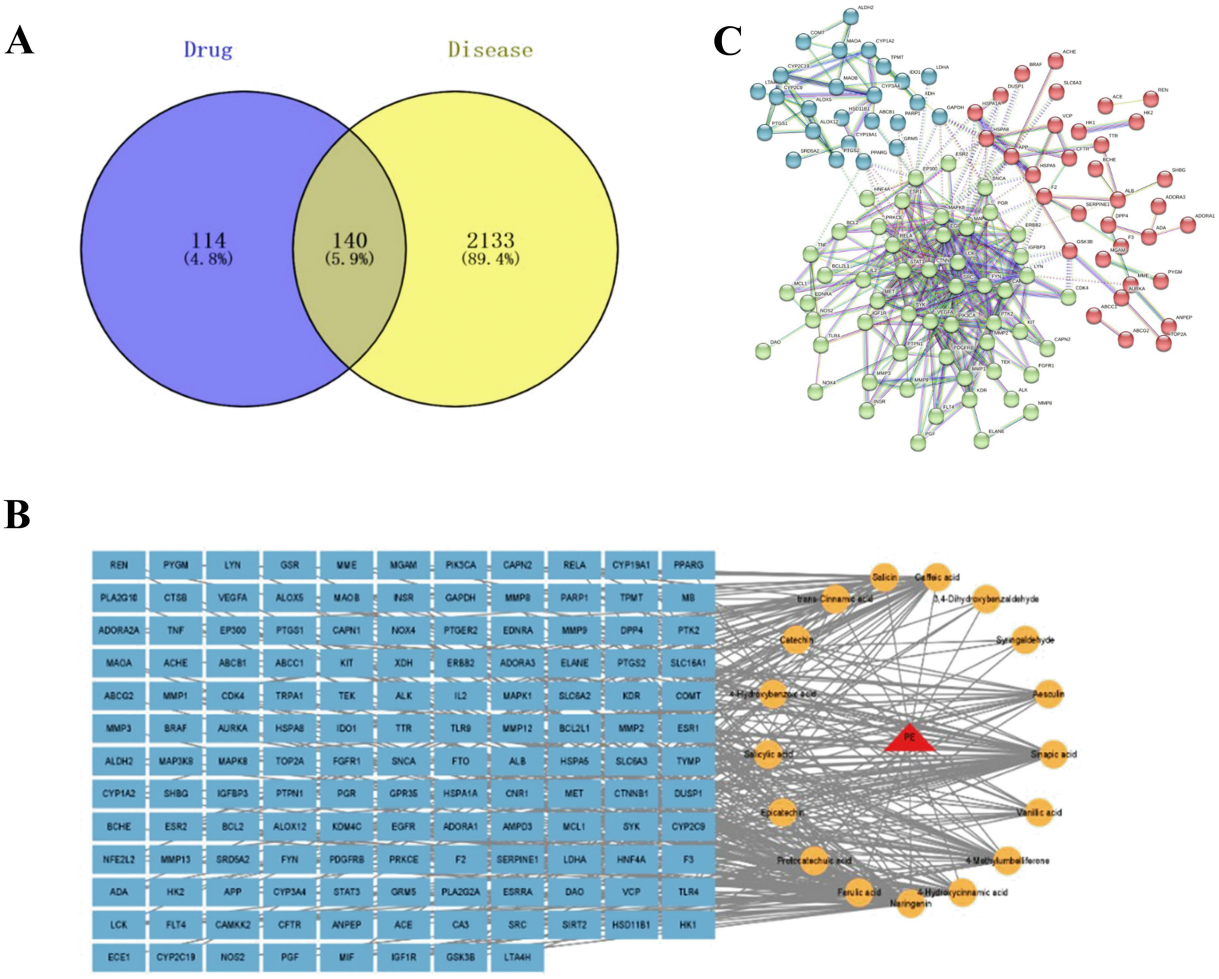
Venn diagram of drug and disease targets **(A)**; fresh-cut pitaya polyphenols – components – common targets **(B)**; mutual mapping of common target PPI **(C)**. Note: The blue circle on the left is the target of fresh-cut pitaya polyphenols, and the yellow circle on the right is the gene target of exercise fatigue **(A)**.

In this experiment, the results of multiple databases were integrated to identify potential anti-fatigue targets for fresh-cut pitaya polyphenol extracts. 2,274 fatigue-related targets were retained. In addition, after removal of ineffective and/or du-plicate targets, 254 potential targets of pitaya polyphenols were identified. The Venny software crossed the fatigue targets of pitaya polyphenols and identified 140 candidate targets with anti-fatigue effects (Figure 1A).

#### 3.1.3 Construction of a network of active ingredients and target points for exercise fatigue in pitaya polyphenols

The regulatory network construction results of fresh-cut pitaya polyphenols and exercise fatigue were shown in Figure 1B. It can be seen from the figure that an active ingredient corresponds to a variety of targets, a target can exist as an application point for a variety of ingredients, and a target can also act through a variety of ways, which indicates that fresh-cut pitaya polyphenols can alleviate exercise fatigue through multi-component, multi-target, multi-pathway, mutual coordination and co-regulation.

#### 3.1.4 Target analysis in PPI network

PPI networks and target cluster analysis have been employed to predict and identify key targets in the pathogenesis of disease. In this study, a confidence level of 0.900 was set, resulting in the formation of a major network comprising interacting genes (Figure 1C). The degree of string-derived relevant nodes was employed to analyze the importance or criticality of proteins. The results demonstrated that certain inflammatory factors, growth factors, cell proliferation and apoptosis-related factors were situated at the core of the network, indicating that these factors played a pivotal role in the anti-fatigue effects of fresh-cut pitaya polyphenols.

#### 3.1.5 Enrichment analysis of GO and KEGG pathways

**Figure 2:**
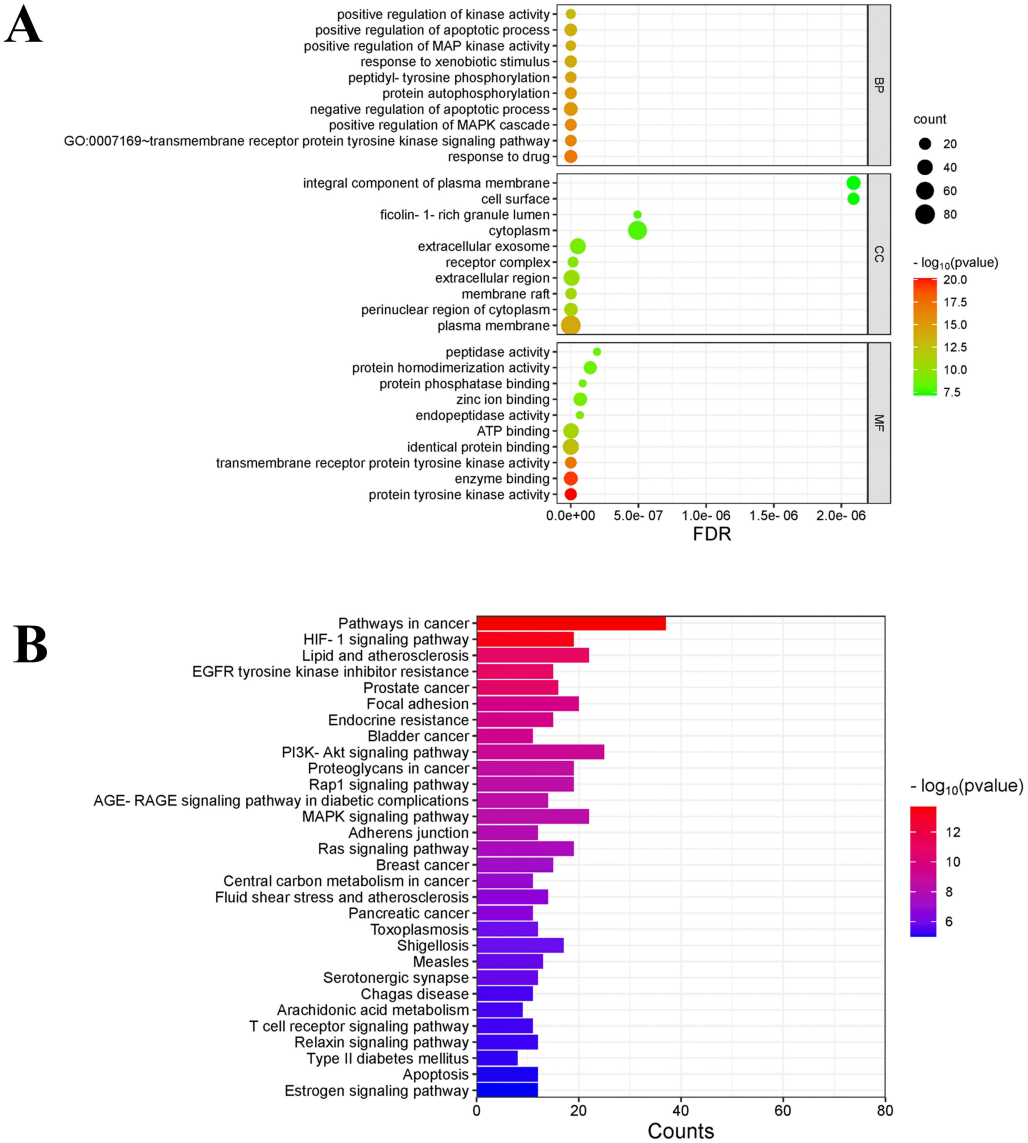
GO analysis and KEGG enrichment analysis.

GO enrichment analysis was performed on 140 potential targets of fresh-cut pitaya polyphenols against exercise fatigue through DAVID database (*p* < 0.01), and a total of 811 items were obtained, including 572 biological processes (BP). There were 87 cellular components (CC) and 152 molecular functions (MP), and the top 10 items of each part were screened according to the *p*-value (Figure 2A). The results showed that the anti-exercise fatigue of fresh-cut pitaya polyphenols involved multiple biological processes. It affects many cell components and molecular functions, such as membrane receptor protein tyrosine kinase signaling pathway, positive regulation of MAPK cascade, protein phosphorylation, negative regulation of apoptosis process, and peptidyl tyrosine phosphorylation.

A total of 160 related signal pathways were obtained by KEGG enrichment analysis (*p* < 0.01). The bubble map of relevant signaling Pathways prepared according to the *p*-value showed that the anti-exercise fatigue of fresh-cut pitaya polyphenols in-volved disease signaling Pathways in cancer, endocrine resistance, Lipid and atherosclerosis, EGFR tyrosine kinaseinhibitor resistance, signal transduction pathway (PI3K/AKT signaling pathway, HIF-1 signaling pathway), etc. (Figure 2B). The SRC family kinases (SRC) and Signal transducer and activator of transcription 3 (STAT3) in the pathway may be emerging drug targets for anti-exercise fatigue therapy. The PI3K-AKT pathway is an intracellular signal transduction pathway that responds to extracellular signals, thereby promoting a number of cellular processes, including metabolism, proliferation, cell survival, growth and angiogenesis (16). In light of the core target and KEGG pathway enrichment results in PPI network cluster analysis, it can be posited that fresh-cut pitaya polyphenols may play an anti-exercise fatigue role by inhibiting the SRC/STAT3 signaling pathway and activating the PI3K/AKT pathway.

#### 3.1.6 Molecular docking results

**Figure 3:**
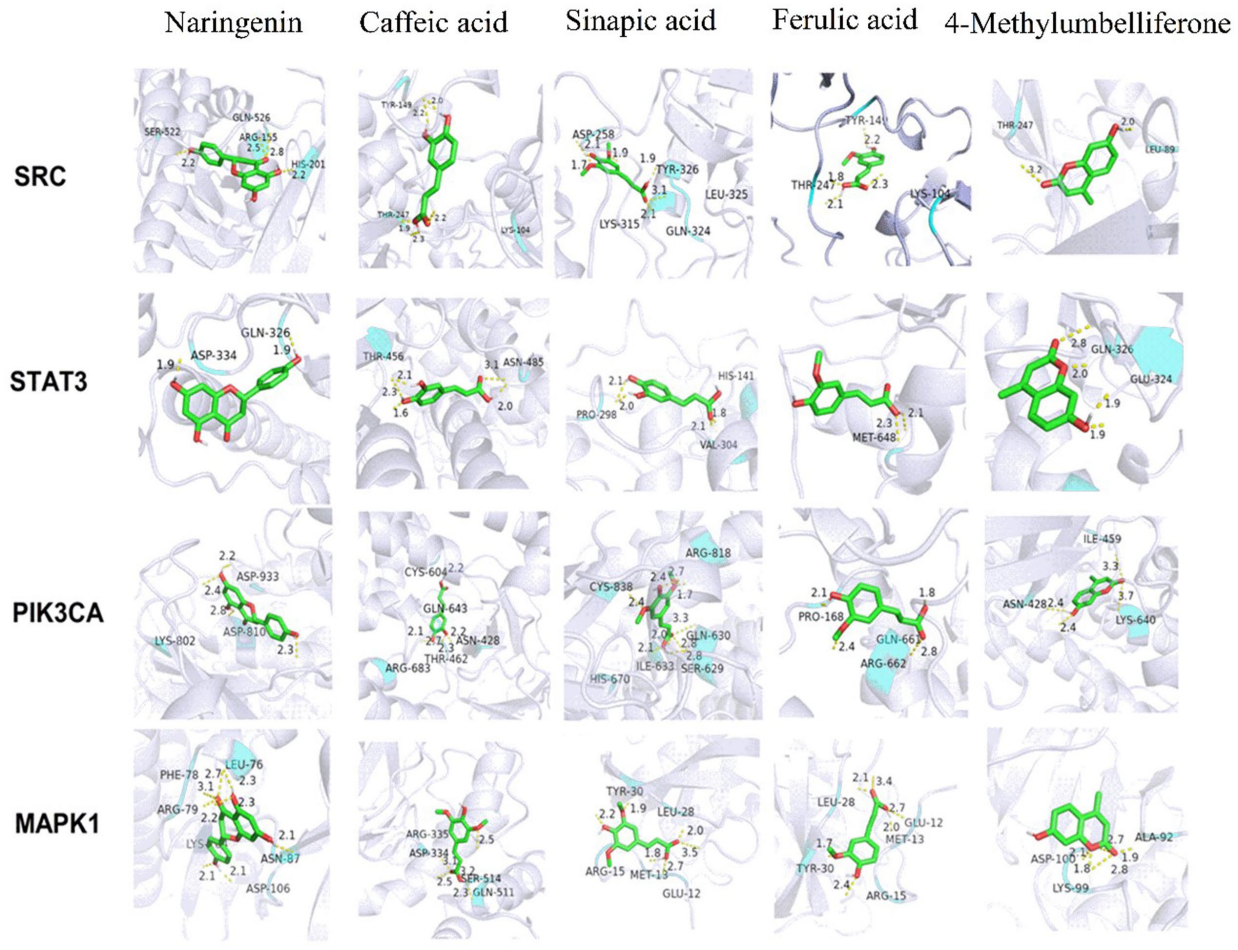
Molecule docking results of key active ingredients and key targets.

Furthermore, the potential for fresh-cut pitaya polyphenols to enter the active pockets of target proteins was predicted, along with the affinity index between them. Molecular docking was conducted on the four proteins (Figure 3). Fresh-cut pitaya polyphenols are situated within the active pockets of target proteins and interact with one another to varying degrees.

Table 1 presents the binding information of fresh-cut pitaya polyphenols to target proteins. The smaller the bond energy, the more readily the interaction can form spontaneously. The results demonstrated that the binding energies were all below −1.2 kcal/mol, and fresh-cut pitaya polyphenols showed favorable binding potential for SRC, STAT3, PIK3CA and MAPK1 targets. It is worth noting that the binding energies of naringenin to SRC, PIKCA and MAPK1 are −6.72 kcal/mol, −6.28 kcal/mol and − 6.32 kcal/mol, respectively, so naringenin may be an effective ingredient in relieving exercise fatigue. Molecular docking results showed that fresh-cut pitaya polyphenols were bound to various amino acid residues through hydrogen bonding interactions, which strongly supports the hypothesis that these key targets play an important role in the anti-exercise fatigue effect of fresh-cut pitaya polyphenols.

**Table 1:** Binding energies of key active ingredients and key targets (kcal/mol).

	Key active compounds				
	Naringenin	Caffeic acid	Sinapic acid	Ferulic acid	4-Methylumbelliferone
SRC	−6.28	−5.72	−4.94	−5.19	−5.57
STAT3	−4.39	−3.73	−2.99	−2.98	−4.44
PIK3CA	−6.28	−4.56	−4.97	−5.33	−5.61
MAPK1	−6.32	−4.28	−4.22	−4.3	−5.46

### 3.2 Pitaya polyphenols increased the exhaustion time of swimming in tired mice and improve physiological indexes

**Figure 4:**
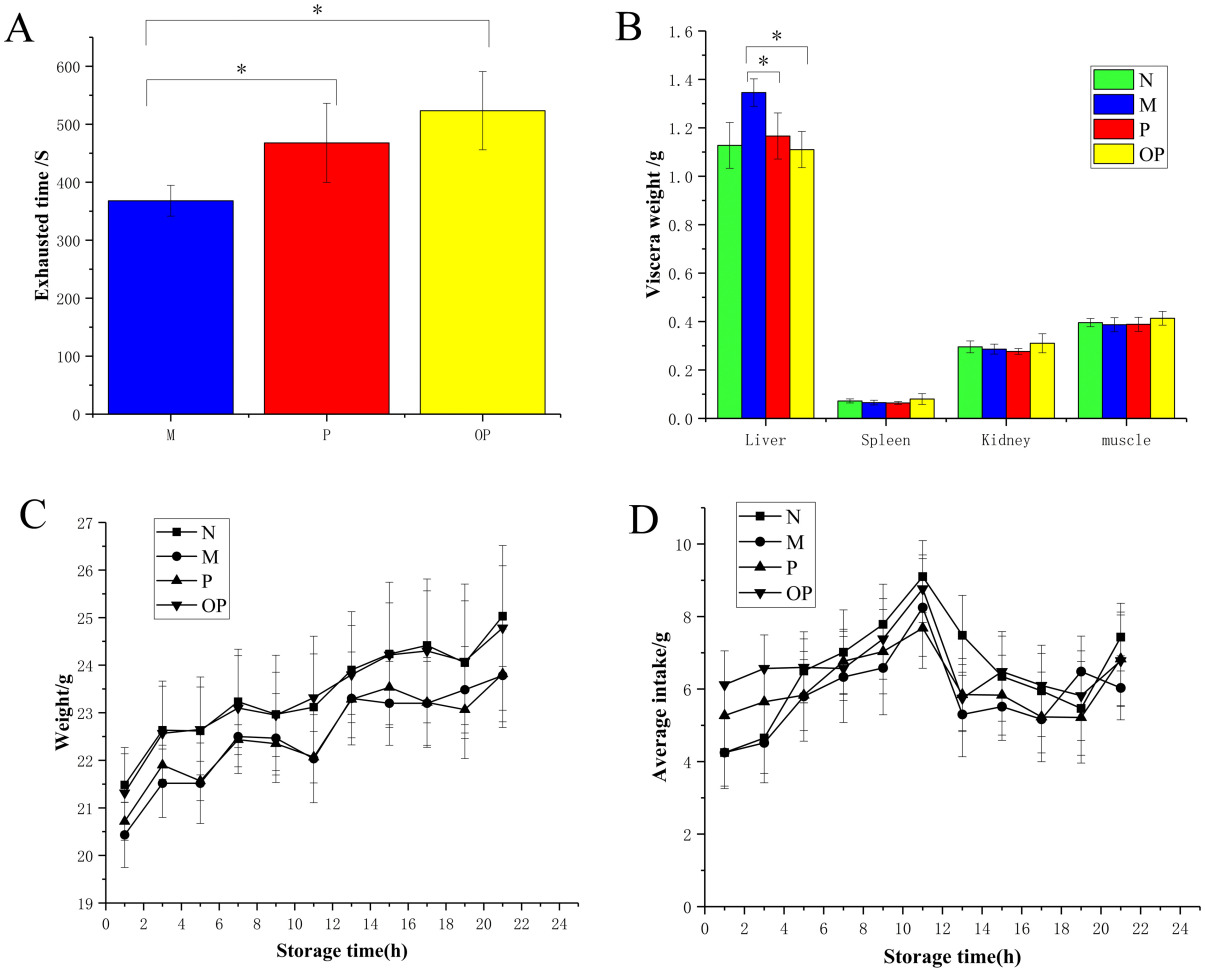
Exhausted time **(A)**, visceral weight **(B)**, body weight **(C)**, and average intake **(D)** of N, M, P, and OP groups.

As illustrated in Figure 4A, mice in the P and OP groups, which were treated with pitaya fruit polyphenols by gavage, exhibited a notable increase in weight-bearing swimming time (*p* < 0.05) when compared to the M group. This prolonged the exhaustion time of the mice swimming. In comparison to the P group, the OP group exhibited a significantly prolonged exhaustion time (*p* < 0.05), from 467.90 s in the P group to 523.40 s in the OP group, representing an increase of 11.86%. The results demonstrated that both P and OP were capable of enhancing the swimming ability of mice, thereby indicating that both P and OP exhibited anti-fatigue effects. The ozone treatment was found to enhance the anti-exercise fatigue potential of fresh-cut pitaya fruit.

There was no significant difference in visceral weight between the P and OP groups compared to the N group, indicating that the dose was appropriate (Figure 4B). Notably, liver and spleen indexes in the P and OP groups decreased significantly to the same level as those in the N group compared to the M group (*p* < 0.05). The results showed that P and OP did not cause significant changes in the internal organs of tired mice. There was no significant change in muscle index in P and OP compared with group M, which may be due to individual differences in the mice and the similar in-tensity of pharmacological action in the P and OP groups, which corresponds to pro-longed exhaustive swimming time in the mice.

During the entire test period, body weight and average food intake did not change significantly in the P and OP groups compared to the N group (*p* > 0.05). As can be seen in Figure 4C, with the increase of modeling time, the body weight of mice in each treatment group showed a normal growth trend. As shown in Figure 4D, there was no significant difference in mean intake between the groups, and there was a brief peak in intake at day 11, followed by a decline to the average level.

### 3.3 Pitaya polyphenols reduced oxidative stress in tired mice

It is evident that the adverse reactions caused by excessive exercise are closely related to the level of oxidative stress. The results demonstrated that groups P and OP could play an anti-fatigue role by increasing the antioxidant level of mice. Furthermore, the ozone-treated fresh-cut pitaya polyphenols (OP group) exhibited a more pronounced anti-exercise fatigue effect. Ozone treatment enhanced the total antioxidant capacity of fresh-cut pitaya polyphenols by 54.17%, accompanied by a 32.8% reduction in MDA content.

**Figure 5:**
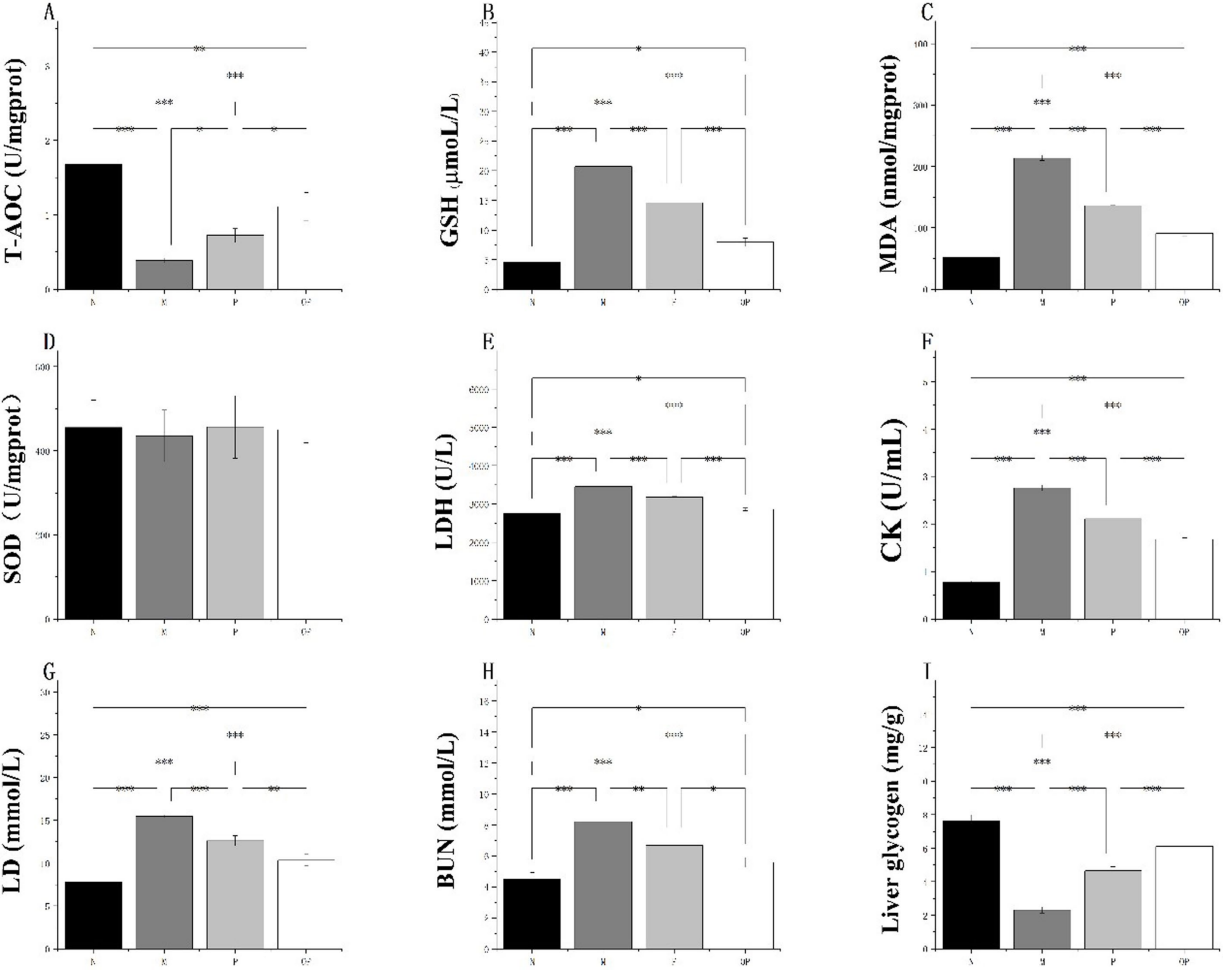
T-AOC **(A)**, GSH content **(B)**, MDA content **(C)**, SOD activity **(D)**, LDH content **(E)**, CK content **(F)**, LD content **(G)**, BUN content **(H)**, and Liver glycogen content **(I)**, of N, M, P and OP groups.

As illustrated in Figure 5A, the antioxidant capacity of skeletal muscle and serum in group M was found to be significantly diminished in comparison to group N, which did not undergo fatigue exercise (*p* < 0.05). This result corroborates the hypothesis that an elevated oxidative stress level can result in adverse effects following fatigue exercise. After supplementation of fresh-cut pitaya polyphenols (P and OP groups) in daily dietary intake, the antioxidant capacity of each group was significantly increased than that of the M group (*p* < 0.01), indicating the anti-exercise fatigue potential of pitaya fruit polyphenols.

It is noteworthy that T-AOC (skeletal muscle) in the P and OP groups increased by 84.61 and 184.62%, respectively, in comparison to the M group (Figure 5A). Furthermore, there was no significant difference between the SOD (Figure 5D) groups. In comparison to the control group (N), the concentration of reduced glutathione (GSH) in the serum of the experimental groups (M, P, and OP) exhibited a significant increase of 357.62, 222.08 and 75.06%, respectively (Figure 5B). In comparison to the M group, the accumulation of MDA in the P and OP groups was found to be significantly reduced (*p* < 0.05). MDA levels in the P and OP groups were reduced by 36.41 and 57.27%, respectively (Figure 5C). The results demonstrated that the P and OP groups exhibited anti-fatigue properties, which were attributed to their ability to enhance the antioxidant capacity of mice. Furthermore, OP group exhibited superior anti-exercise fatigue properties.

### 3.4 Pitaya polyphenols improved substrate metabolism accumulation in tired mice

The serum CK level of mice is a direct clinical biomarker that reflects muscle discomfort or even damage after exercise fatigue. The lower the CK level, the stronger the recovery ability after exercise injury. LD is an adverse product of intense exercise in a short period of time and is also a cause of fatigue and discomfort in the body. Rapid removal of lactic acid is conducive to relieving fatigue. BUN is a bad blood waste after sports injury, usually the higher the content, the higher the degree of fatigue and even damage of the body. Therefore, by evaluating the activities of CK and LDH in serum and the contents of LD and BUN, we can see the protective effect of pitaya fruit polyphenols on tissue damage caused by exercise fatigue in mice.

A comparison of the data from group N with that from group M revealed that the levels of LDH, CK, LD and BUN were significantly elevated in the latter group (*p* < 0.05 or *p* < 0.01) (Figures 5E–H). In comparison to the control group, the levels of LDH, CK, LD and BUN were significantly reduced in the pitaya fruit polyphenol administration groups (*p* < 0.05). In particular, CK in the P and OP groups was reduced by 23.55 and 39.49%, respectively, BUN by 18.26 and 31.80%, and LDH by 8.03 and 17.25%, respectively. These findings indicate that supplementation of the P and OP groups can effectively maintain substrate metabolism in mice with exercise fatigue and alleviate fatigue-related damage.

### 3.5 Pitaya polyphenols improved energy metabolism in tired mice

After swimming, the glycogen content in group M was significantly decreased compared with group N (*p* < 0.05). The glycogen content of groups P and OP was significantly increased (*p* < 0.05). Compared with the glycogen content of group M, the liver glycogen content of group P and OP was increased by 1.0 and 1.6 times, respectively. Compared with group M, there was a significant difference between group P and group OP (*p* < 0.05) (Figure 5I). In addition, compared with mice without high-intensity swimming (0.3 ± 0.03 mg/g), the level of muscle glycogen in blood of mice in exercise fatigue control group after exhaustive swimming training was significantly decreased (0.23 ± 0.01 mg/g), *p* < 0.01; However, different pitaya fruit polyphenol intragastric exercise groups (P group and OP group) can improve the level of muscle glycogen, the statistical results are: 0.28 ± 0.06 mg/g and 0.52 ± 0.05 mg/g, respectively, and the content of muscle glycogen in OP group is significantly higher than that in P group, *p* < 0.01. The above results indicated that fresh-cut pitaya polyphenols could increase the glycogen reserve of tired mice and had anti-fatigue effect. Ozone treatment increased the liver glycogen content of pitaya polyphenols by 85.71%.

### 3.6 Pitaya polyphenols improved skeletal muscle pathology

**Figure 6:**
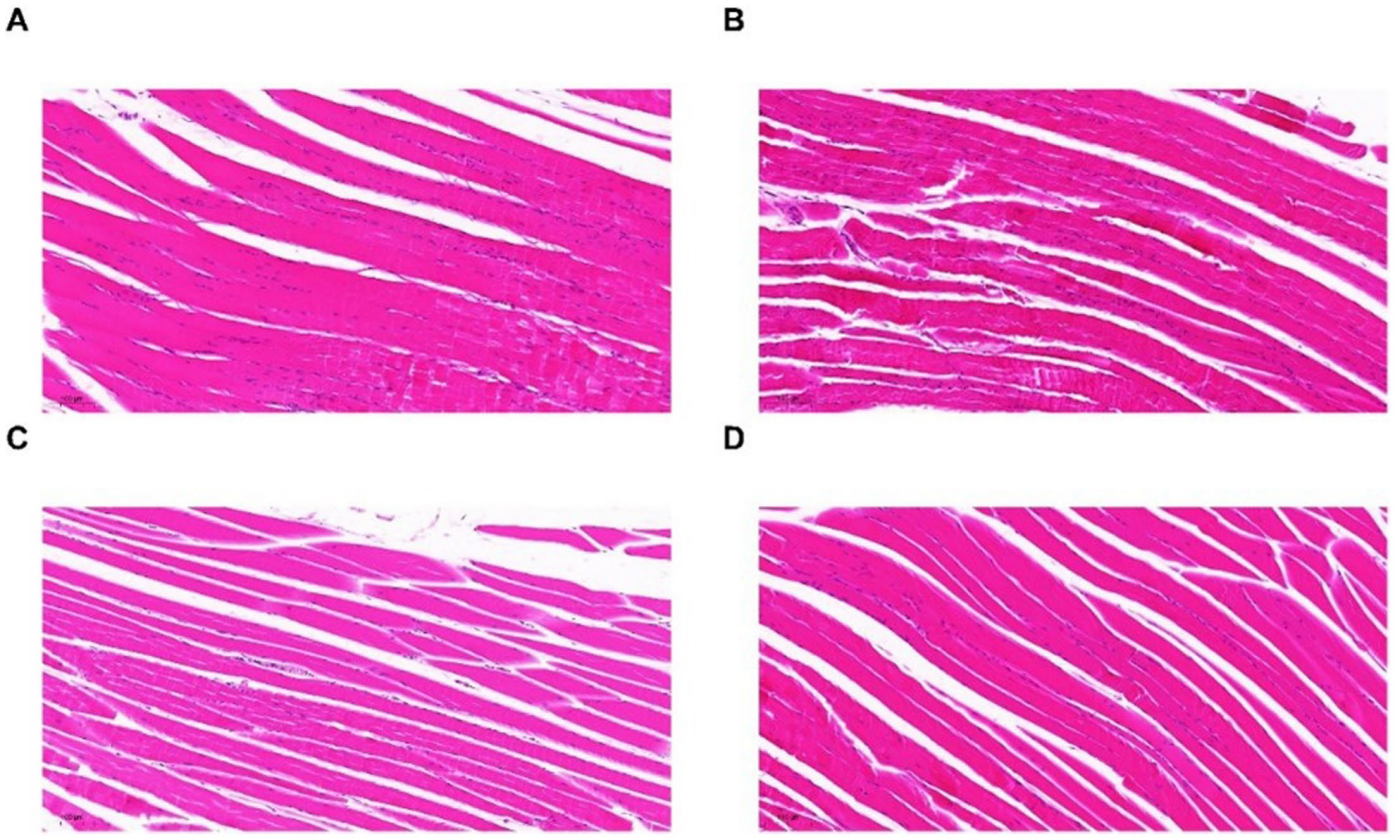
HE images of skeletal muscles in N group **(A)**, M group **(B)**, P group **(C)**, and OP group **(D)**.

Observation of HE-stained sections of mice in each group revealed that myocytes in the muscle tissues of mice in group N were neatly arranged, orderly distributed, and structurally normal. Furthermore, no evidence of inflammatory cell infiltration or myofiber hyperplasia was observed (Figure 6A), indicating that the muscle cells were healthy. However, following strenuous exercise, skeletal muscles of mice in group M exhibited nuclear aggregation, myofiber necrosis, degeneration, and inflammatory cell infiltration (Figure 6B). This indicated that the muscle cells were in a poor state due to high-intensity exercise. After nutritional supplement (pitaya polyphenols), muscle necrosis and muscle fiber degradation in groups P and OP were improved compared with group M, and the OP group had the fewest inflammatory cells (Figures 6C,D). Therefore, ozone treatment enhanced the anti-fatigue potential of fresh-cut pitaya polyphenols. It can be concluded that pitaya polyphenols have a certain intervention effect on the exercise fatigue of mice, and due to the higher content of polyphenols in the OP group of pitaya fruit after ozone treatment, it showed better anti fatigue effect.

### 3.7 Pitaya polyphenols reduced fatigue in mice through PI3K/AKT and SRC/STAT3 pathways

**Figure 7:**
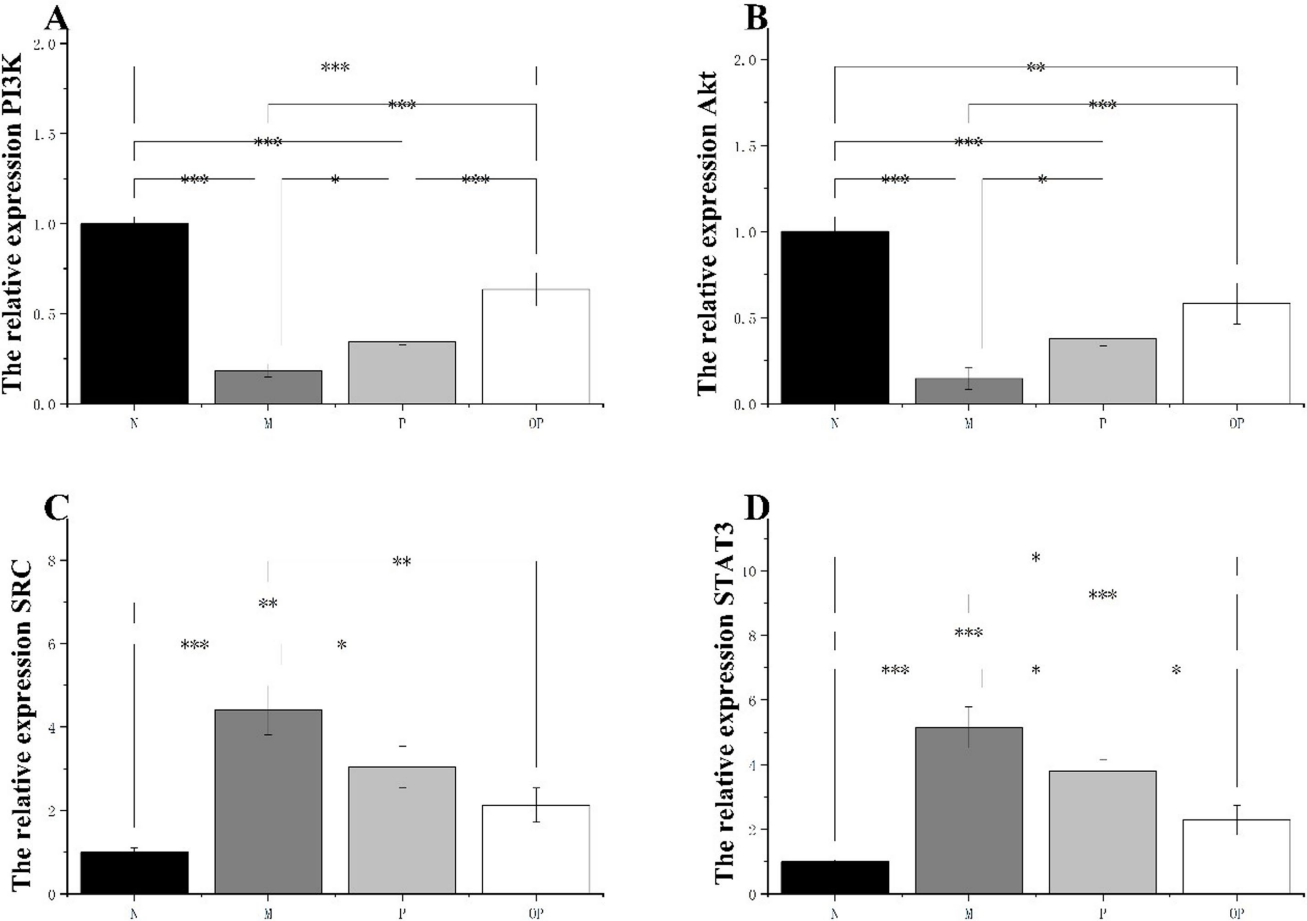
The expression of PI3K, Akt, SRC and STAT3 was assessed by RT-qPCR.

In comparison to group N, the mRNA expression levels of PI3K and AKT in the muscle tissue of group M were found to be significantly reduced (*p* < 0.05). This reduction was observed to be 82.00 and 85.00% for PI3K and AKT, respectively. In comparison to group M, the mRNA expression levels of AKT and PI3K in groups P and OP were found to be significantly elevated (*p* < 0.05) (Figures 7A,B). This suggests that groups P and OP may enhance the body’s anti-fatigue capacity by activating the PI3K/AKT pathway.

A comparison of the mRNA expressions of SRC and STAT3 in group M with those of group N revealed a significant increase in the former (*p* < 0.05). It is possible that the observed increases in the expression of SRC and STAT3 mRNA are a consequence of severe heart failure resulting from excessive exertion. In comparison to the pitaya fruit polyphenol administration group, the mRNA expressions of SRC and STAT3 were found to be significantly decreased (*p* < 0.05) (Figures 7C,D). In comparison to the P group, the expression of SRC and STAT3 in the OP group was found to be down-regulated (*p* < 0.05). Furthermore, the down-regulated effect in the OP group was found to be more significant than that in the P group (*p* < 0.05). The findings demonstrated that the anti-fatigue efficacy of the P and OP groups could be enhanced by the inhibition of the SRC/STAT3 pathway.

### 3.8 Pitaya polyphenols improved intestinal flora diversity in tired mice

**Figure 8:**
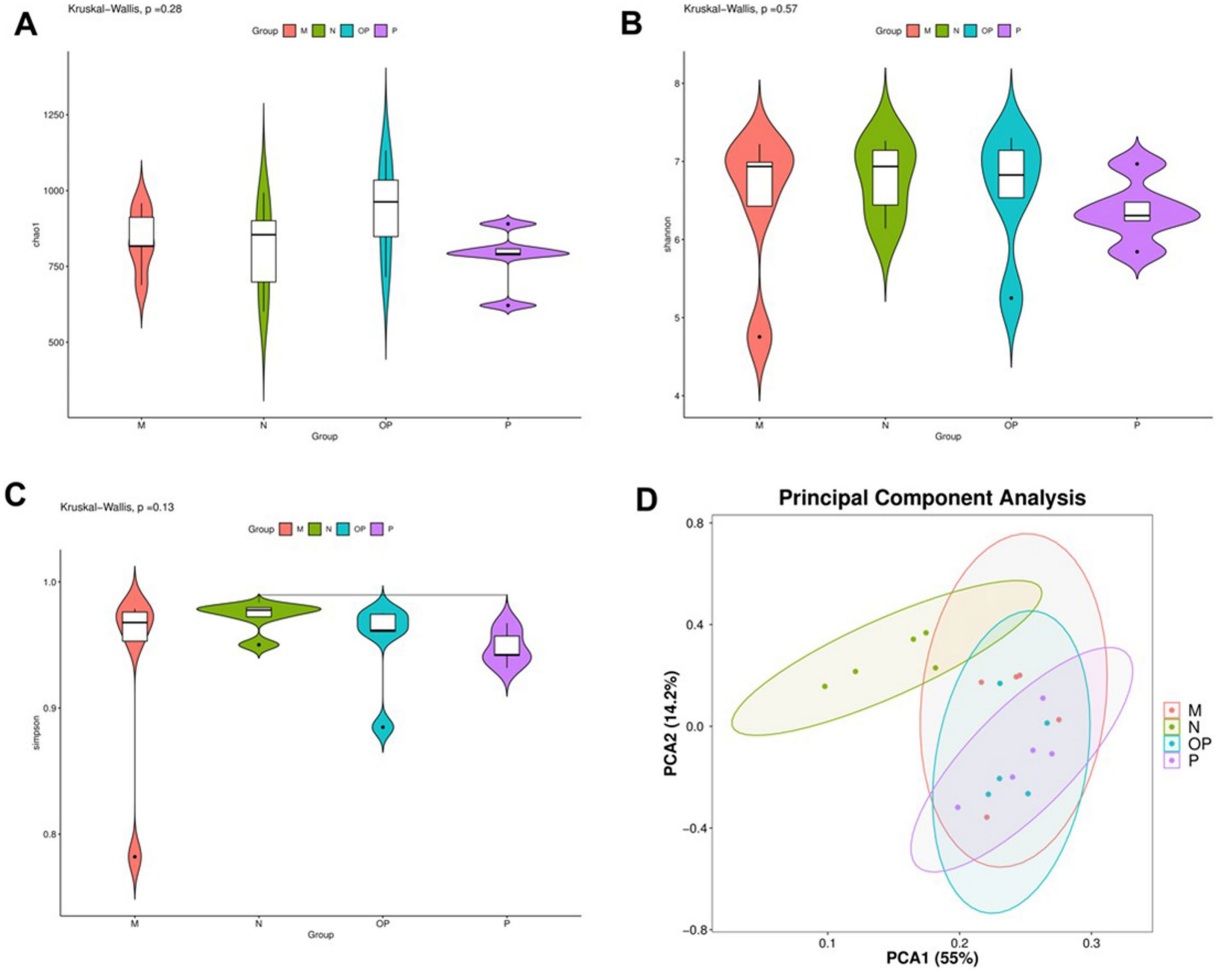
Diversity analysis results of gut microbiota in N, P, and OP groups.

The results of Alpha diversity analysis are shown in Figure 8. The analysis of microbial diversity revealed no significant differences in the data among the groups (*p* > 0.05). The beta diversity analysis revealed significant species-level differences among the various subgroups. At the generic level, the contribution rate of the first principal component to the different species was 55%, and that of the second principal component to the different species was 14.2%, with a total contribution rate of 69.2%. Group N exhibited a notable divergence from the other groups, as did the beta diversity (*p* < 0.05). These findings suggest that the intestinal microbiome of swimming fatigue mice is markedly distinct from that of the normal control group, and that indicator species can be identified through differences in microbial composition.

**Figure 9:**
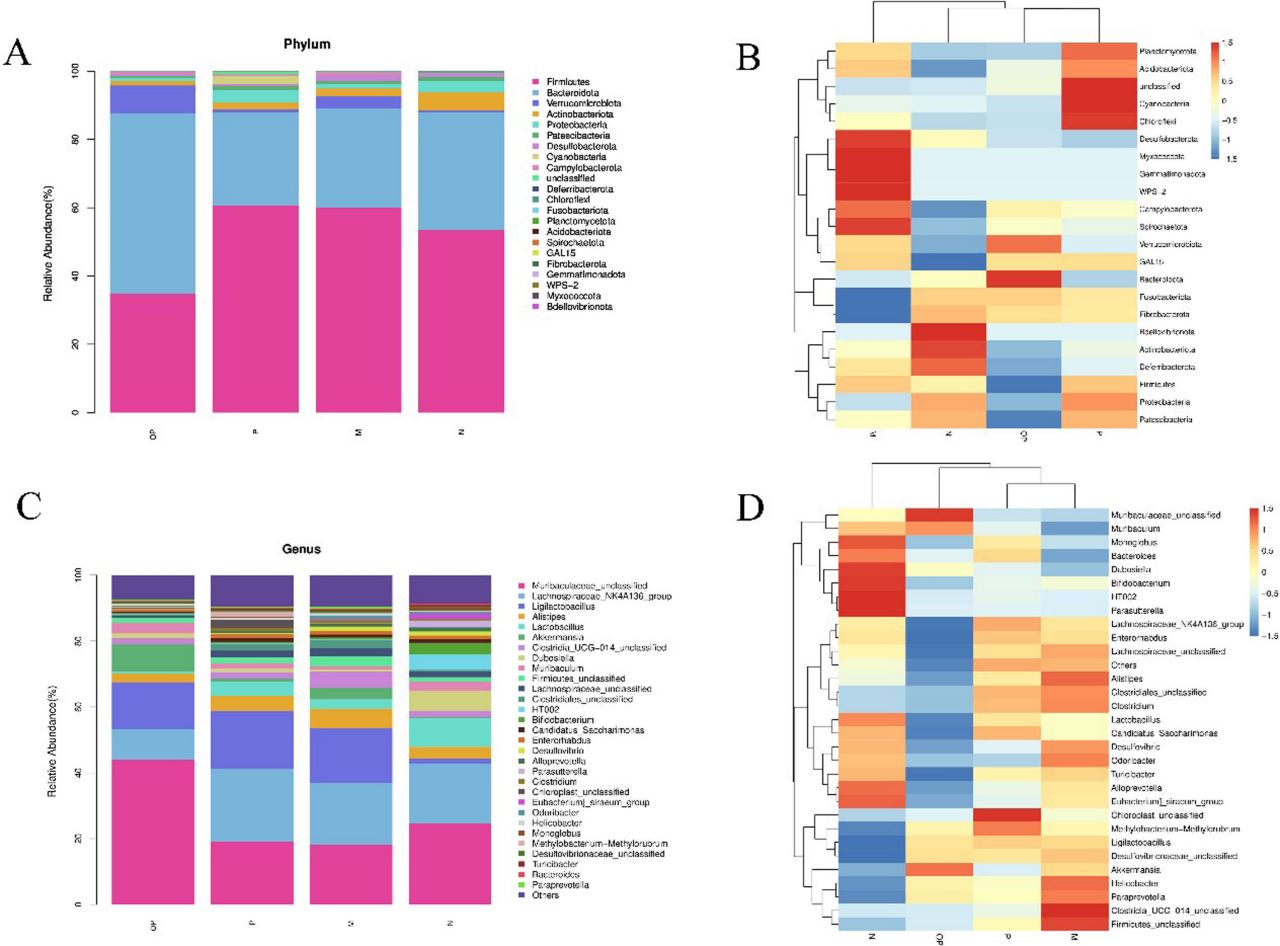
Stacking diagram and heat map of relative abundance of microorganisms at the phylum level **(A, B)**, and at the genus level **(C, D)**.

As illustrated in Figure 9, the intestinal flora in the cecum of mice was predominantly composed of Firmicutes (F), Bacteroidetes (B), Verrucomicrobia, and Actinobacteria. In the control group (group N), model group (group M), and the group receiving the polyphenol-rich extract (group P), the most prevalent microorganisms were Firmicutes, followed by Bacteroidetes. The relative abundance of microbes in the OP treatment group differed from that observed in the other three groups. Bacteroidetes was the most dominant microorganism, followed by Firmicutes. As illustrated in Figure 9, the F/B ratio of mice in the fatigue group (group M) was found to be elevated in comparison to the normal group (group N) (*p* < 0.05). This trend was observed to be attenuated by the administration of fresh cut pitaya fruit polyphenols following ozone treatment.

In terms of heat maps, at the genus level (Figures 8C,D), compared to group N, Ligilactobacillus, Alistipes, Akkermansia, Odoribacter, Desulfovibrionaceae unclassified, Paraprevotella, and Clostridia_UCG-01 in group M 4_unclassified was significantly increased (*p* < 0.05), (Lactobacillus, Monoglobus, Eubacterium)_siraeum_group, Parasutterella, HT002 and Bifidobacterium were significantly decreased (*p* < 0.05). In comparison to group M, the levels of *Helicobacter pylori*, Desulfovibrio and Eubacteria in groups P and OP were also significantly reduced, indicating that groups P and OP may be effective in reducing the level of body inflammation and infection in tired mice. In the P and OP groups, methyl rhodobacter and Ligerella exhibited a downregulation effect. However, this was less pronounced in the P group, while the OP group displayed a more pronounced downregulation effect. This suggests that the OP group may have a superior ability to inhibit the proliferation of adverse microorganisms and alleviate exercise fatigue.

**Figure 10:**
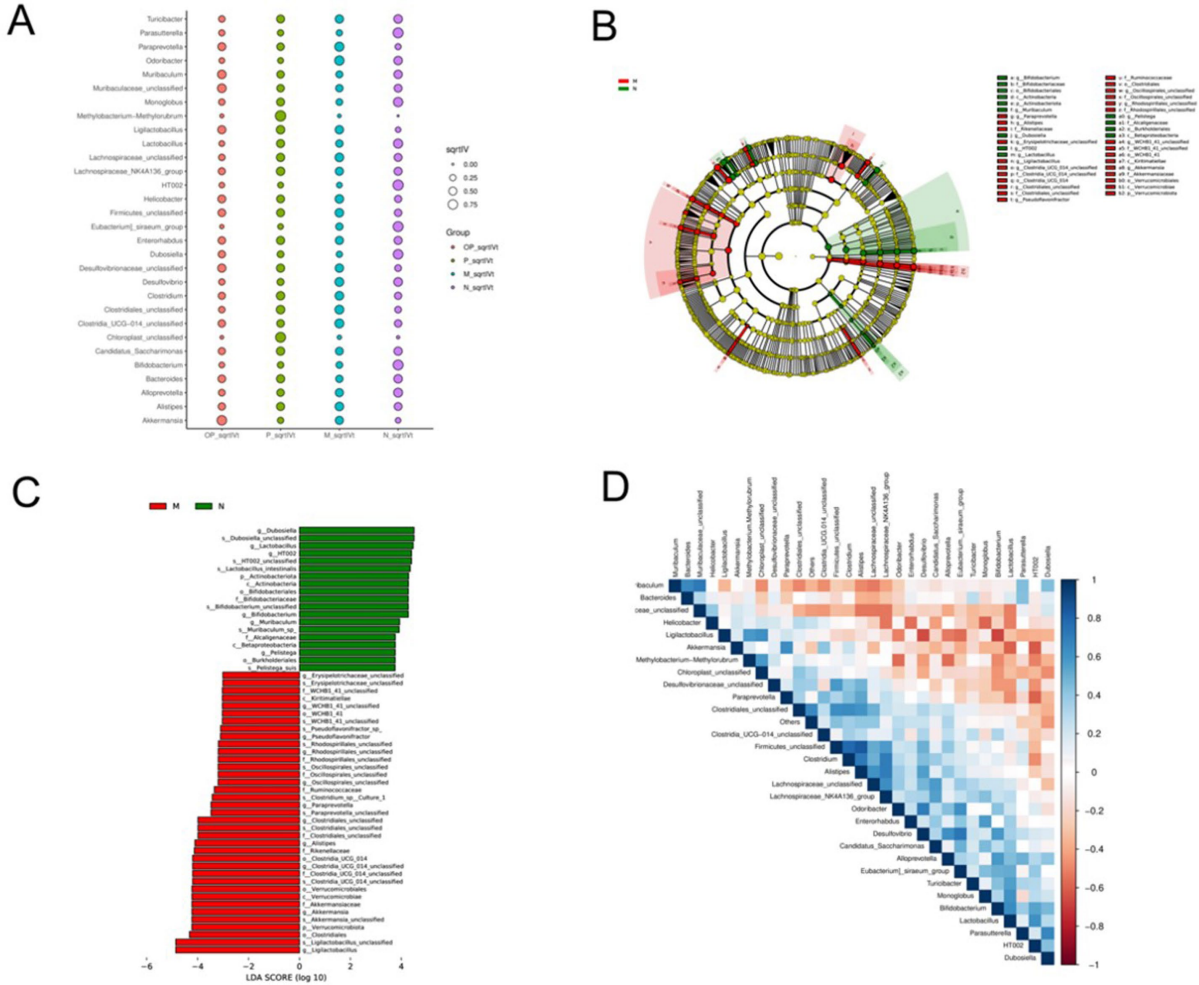
Indicator species analysis of gut microbiota in each group **(A)**, LefSe difference analysis of M and N groups **(B, C)**, and correlation analysis heatmap of microorganisms **(D)**.

The primary objective of LefSe analysis is to contrast two or more groups and identify the species (biomarker) exhibiting a significant disparity in abundance be-tween the groups, a methodology that has been extensively employed (17). As shown in Figure 10, the greater the sqrtIVt value, the better the species is as an indicator spe-cies for the treatment group. The possible biomarkers in groups M, N, P and OP were Odoribacter, HT002, Methylobacteria-methylorubrum and Akkermansia, respectively.

## 4 Discussion

It is generally believed that exercise is beneficial to health, nevertheless, excessive exercise has been demonstrated to cause damage to organs and tissues, as well as fatigue. In previous studies designed to assess exercise fatigue, forced swimming tests were frequently employed due to their objectivity, effectiveness and convenience (18). Therefore, in this study, we established a mouse swimming fatigue model. We found that fresh-cut pitaya fruit polyphenols can alleviate exercise-induced fatigue. No significant differences were observed in body weight or organ index between the polyphenol and control groups, indicating the high safety of fresh-cut pitaya fruit polyphenols. Studies have shown that during intense exercise, muscle cells will be adversely affected by inflammatory cells, causing fibrocytes to dissolve and produce cracks (19); However, the addition of fresh-cut pitaya fruit polyphenols reduced the occurrence of such cracks. The above results all reflect the anti-exercise fatigue potential of fresh cut pitaya fruit polyphenols.

During exercise, the body’s metabolism is elevated, resulting in increased oxy-gen consumption. This, in turn, leads to the generation of a significant number of free radicals, Strenuous exercise produces excessive free radicals, disrupting the REDOX balance in the body, resulting in increased MDA levels (20, 21). High MDA concentrations are considered to be a major factor in triggering physical fatigue8. The MDA level of the model group in this experiment was found to be significantly higher than that of the other treatment groups (*p* < 0.05), indicating that the subjects had experienced exercise-induced body fatigue. The supplementation of fresh-cut pitaya fruit polyphenols was observed to reduce MDA levels in the liver and muscle, thereby suggesting a potential role in the reduction of fatigue. SOD is a well-known antioxidant enzyme that plays a pivotal role in maintaining equilibrium between oxidation and antioxidant activity within the body. Ozone-treated fresh-cut pitaya fruit polyphenols have been demonstrated to significantly increase SOD activity in liver and muscle tissue, indicating that ozone treated fresh-cut pitaya polyphenols can reduce fatigue through antioxidant effect.

Energy consumption, metabolite accumulation and oxidative stress are the broad causes of exercise-induced fatigue (22, 23). The body’s adequate energy reserves and good supply capacity are crucial for maintaining healthy exercise. Therefore, the storage of glycogen in the muscles and liver is of particular importance for improving endurance. Experiments have shown that tea polyphenols can reduce the expression of glycogen synthase kinase (GSK-3βm) RNA and increase the expression of glycogen synthase 1 (Gys1), thereby reducing substrate consumption caused by exercise fatigue (7). The experimental results in this chapter show that ozone treatment can improve the effect of fresh-cut pitaya polyphenols on relieving exercise-induced fatigue *in vivo* by reducing the accumulation of lactic acid and blood urea nitrogen.

There is growing evidence that there is a strong link between human health diseases and the gut microbiome (24, 25). In a recent study, Yu (9) prepared hawthorn polyphenol microcapsules (HPM) by microencapsulation and demonstrated their potential mechanisms of action in mice exhibiting fatigue. These mechanisms included the regulation of the AMPK signaling pathway and the maintenance of equilibrium within the intestinal flora. Following treatment with HPM, the weight-bearing swimming ability of tired mice was found to have increased significantly (*p* < 0.05), accompanied by enhanced antioxidant capacity (*p* < 0.05). Intestinal microbiota metabolites can provide energy to liver and muscle cells, thereby regulating energy metabolism during exercise. Furthermore, excessive exercise has been demonstrated to alter the diversity and abundance of gut flora. In this experiment, the supplementation of fresh-cut pitaya fruit polyphenols was observed to increase the relative abundance of Bacteroides in the cecum and restore the damage to the intestinal flora caused by intense exercise, in comparison to the model group. The results of the gate-level analysis demonstrated that exercise fatigue was associated with an increase in the F/B ratio of the cecum, which is in accordance with previous findings (26). The supplementation of pitaya fruit polyphenols decreased the F/B ratio to varying degrees. Considering the diversity of flora, fresh-cut Pitaya polyphenols maintained *α* and *β* diversity of flora. Therefore, fresh-cut pitaya fruit polyphenols can alleviate exercise-induced fatigue by balancing intestinal flora.

## 5 Conclusion

In conclusion, we found that fresh-cut pitaya polyphenols treated with ozone had a better anti-fatigue effect. By increasing the glycogen storage, reducing oxidative stress, and regulating the substrate metabolism to protect skeletal muscle and increase exercise endurance performance. It was found that fresh-cut pitaya polyphenols may be involved in activating PI3K and Akt regulatory factors, balancing intestinal flora, and playing an anti-fatigue role. However, further research is needed to confirm the potential mechanism by which fresh-cut pitaya exerts its anti-fatigue effects. In addition, the beneficial effects of ozone treatment on plant polyphenol content and function still need to be screened and validated in more fruits and vegetables.

## Data Availability

The original contributions presented in the study are included in the article/Supplementary material, further inquiries can be directed to the corresponding author.

## Footnotes

^1^ http://www.swissadme.ch/

^2^ https://pubchem.ncbi.nlm.nih.gov/

^3^ http://www.swisstargetprediction.ch/

^4^ https://sea.bkslab.org/

^5^ https://www.genecards.org/

^6^ https://www.omim.org/

^7^ https://string-db.org

^8^ https://pubchem.ncbi.nlm.nih.gov/

^9^ http://www.swissdock.ch/

^10^ https://www.microbiomeanalyst.ca/

^11^ https://huttenhower.sph.harvard.edu/galaxy
