# Supplementary material for: Ozone treatment regulated the anti-exercise fatigue effect of fresh-cut pitaya polyphenol extracts
Source: Front Nutr. 2024 Nov 25;11:1500681. doi: 10.3389/fnut.2024.1500681 (PMC11625569; doi:10.3389/fnut.2024.1500681)
Supplement: Supplementary file 1 [file Table_1.DOCX]

**Table S1 Active components of fresh-cut pitaya polyphenols**

| Compound name | Formula | Structure | Bioavailability Score |
| --- | --- | --- | --- |
| trans-Cinnamic acid | C_9_H_8_O_2_ | 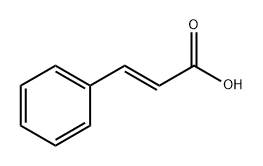 | 0.85 |
| Vanillic acid | C_8_H_8_O_4_ | 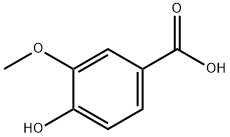 | 0.85 |
| 4-Hydroxybenzoic acid | C_7_H_6_O_3_ | 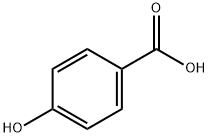 | 0.85 |
| Salicylic acid | C_7_H_6_O_3_ | 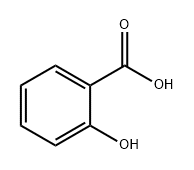 | 0.85 |
| 4-Hydroxycinnamic acid | C_9_H_8_O_3_ | 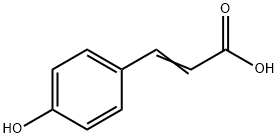 | 0.85 |
| Ferulic acid | C_10_H_10_O_4_ | 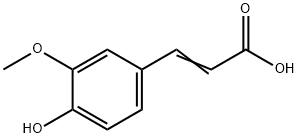 | 0.85 |
| Sinapic acid | C_11_H_12_O_5_ | 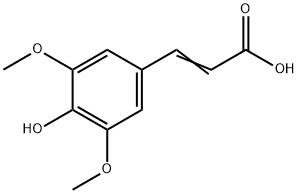 | 0.85 |
| Protocatechuic acid | C_7_H_6_O_4_ | 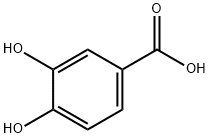 | 0.56 |
| Caffeic acid | C_9_H_8_O_4_ | 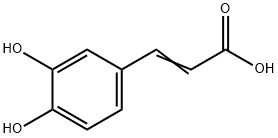 | 0.56 |
| 3,4-Dihydroxybenzaldehyde | C_7_H_6_O_3_ | 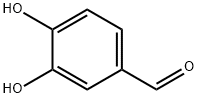 | 0.55 |
| Catechin | C_15_H_14_O_6_ | 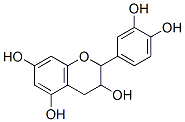 | 0.55 |
| Epicatechin | C_15_H_14_O_6_ | 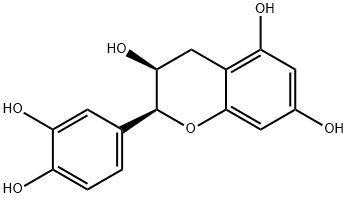 | 0.55 |
| Syringaldehyde | C_9_H_10_O_4_ | 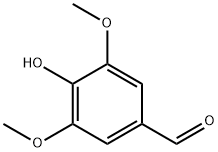 | 0.55 |
| Salicin | C_13_H_18_O_7_ | 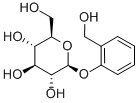 | 0.55 |
| Naringenin | C_15_H_12_O_5_ | 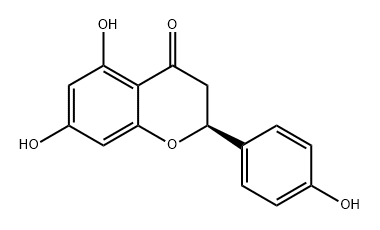   \|  \| \| --- \| | 0.55 |
| Aesculin | C_15_H_16_O_9_ | 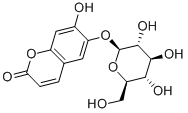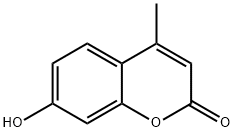 | 0.55 |
| 4-Methylumbelliferone | C_10_H_8_O_3_ |  | 0.55 |
